# Characteristics of Loads of Cattle Stopping for Feed, Water and Rest during Long-Distance Transport in Canada

**DOI:** 10.3390/ani4010062

**Published:** 2014-03-05

**Authors:** Hannah E. Flint, Karen S. Schwartzkopf-Genswein, Ken G. Bateman, Derek B. Haley

**Affiliations:** 1 Department of Population Medicine, Ontario Veterinary College, University of Guelph, 50 Stone Rd E., Guelph, ON, N1G 2W1, Canada; E-Mails: flinth@uoguelph.ca (H.E.F.); kbateman@uoguelph.ca (K.G.B.); 2 Agriculture and Agri-Food Canada, Lethbridge, AB, T1J 4B1, Canada; E-Mail: karen.genswein@agr.gc.ca

**Keywords:** long-distance transport, transportation, cattle, welfare, rest

## Abstract

**Simple Summary:**

This study was designed to benchmark the characteristics of loads of cattle stopping for feed, water and rest during long distance transport in Canada. Another objective of this study was to determine how well these loads were following current Canadian regulations for the length of time animals can spend in transit, and how long they must be rested for. The majority of loads stopping for feed water and rest were transporting cattle to feedlots rather than processing plants. All loads were under the 48 hour maximum allowable time in transit defined under the Canadian transport regulations and all loads exceeded the minimum duration of 5 hours required for feed, water and rest.

**Abstract:**

This study is the first comprehensive examination of long-haul cattle being transported across Canada and off-loaded for feed, water and rest. A total of 129 truckloads were observed at one of two commercial rest stations near Thunder Bay, Ontario. Data collected included information regarding the truck driver, the trailer, the trip, the animals and animal handling. The majority of the loads stopping were feeder calves (60.94%) while 21.09% were weaned calves, and the remaining 14.84% were market weight cattle. The truck loads surveyed were in transit for, on average, 28.2 ± 5.0 hours before stopping and cattle were rested for an average of 11.2 ± 2.8 hours. These data suggest that loads stopping at the rest station were adhering to the regulations stated in the Health of Animals Act, which outline a maximum of 48 hours in transit before a mandatory stop of at least 5 hours for feed, water and rest. There was a large amount of variability around how well recommendations, such as stocking density were followed. Further research is required to assess how well cattle are coping with long-distance transport under current regulations and industry practices.

## 1. Introduction

A census conducted by the Ontario Ministry of Agriculture and Food (OMAF) in 2012 found that over 600,000 cattle were slaughtered at federally and provincially inspected processing plants within the province, this despite a provincial beef herd population of only 321,000 cows [1]. Thus, large proportion of the beef cattle finished for slaughter in Ontario come from out of province, and most come from western Canada, arriving by road transport.

The transportation of livestock within Canada is federally regulated by the Health of Animals Act, which is the enabling Statute of the Health of Animals Regulations [2]. These regulations cover, among other items, definitions for animals that are unfit for transport, facility requirements for loading and unloading, specifications for the vehicle/container, and requirements for the provision of feed and water en route. These regulations state that weaned cattle cannot be confined on the truck for more than 48 hours without being unloaded for a minimum of 5 hours for feed, water and rest somewhere along their journey. The time in transit can be extended to 52 hours if the cattle are expected to reach their final destination within that time. [2].

Several aspects of long distance transport can impact the well-being of the animals. The experience and skill of the truck driver can have a large impact on cattle welfare both through their handling of the cattle at loading (and unloading) and via their actual driving [3]. As loading and unloading have been suggested to be the most stressful portion of transportation [4], good handling practices are important. Transport of longer duration has also been shown to result in animals suffering more injuries and greater weight loss, or shrink, and time in transit can also affect morbidity once the cattle reach their destination [5,6,7]. Stocking density is another factor that can impact welfare during transport. High stocking densities have been shown to limit the animals’ ability to position themselves in their preferred orientation of either parallel or perpendicular to the direction of travel, and this can lead to more animals losing their balance and struggling to maintain footing [8]. This may contribute to the increase in injuries, such as bruises, in cattle transported at high stocking densities [8].

The number and variety of potential welfare concerns arising from long-distance transport inevitably raise the question about the well-being of cattle being transported long distances in Canada, given current industry practices. As a first step to answering this question, the present project was initiated to benchmark the characteristics of loads of cattle stopping for rest as part of their long-distance journey and to also document the practice of providing opportunity for rest.

## 2. Experimental Section

### 2.1. Information Gathered

All methods used in this study were approved by the Animal Care Committee (#12R038) and Research Ethics Board (#12SE009) at University of Guelph. A survey was administered on a voluntary basis and in an interview format to 104 truck drivers who had stopped at a commercial cattle rest station near Thunder Bay, Ontario between April 23, 2012 and June 13, 2012 (spring), and between September 27, 2012 and December 13, 2012 (fall). To collect information on truckloads arriving during the summer months, between June and September, surveys were left available at the rest station for completion by willing drivers, providing information about an additional 25 loads for a total of 129 completed surveys.

When administered in person, the survey was completed immediately after the cattle had been unloaded. The survey was a modified version of the survey used by González *et al.* [9], with questions added to describe the characteristics of the cattle being transported. The survey was designed to collect information regarding five general aspects of cattle transport: the driver, the trailer, the trip, the animals, and the animal handling (Table 1).

**Table 1:** Information that was collected through administering a survey to truck drivers and observing the unloading and reloading of cattle from trucks stopped for feed, water and rest near Thunder Bay, ON (TB).

Driver	Trailer	Trip	Animal	Animal Handling
Sex	No. axles	Origin type	Space allowance	Time to unload
Years of experience	No. compartments	Destination type	No. animals on load	Time to load
CLT ^1^ training (Y/N)	Bedding condition	Distance travelled to TB	Type	Slips unload
	Weather boards	Total distance travelled	Weight class	Falls unload
		Time in transit to TB	Sex	Slips load
		Time at rest station	No. compromised	Falls load
		Weather at unloading	No. down	Carry cattle prod (Y/N)
		Weather during trip	No. dead	
			Lying down in truck	

^1^ Canadian Livestock Transport certification program.

#### 2.1.1. Driver Characteristics

Information collected regarding driver skill included the number of years’ experience hauling cattle and whether they had taken Canadian Livestock Transport (CLT) certification program, a voluntary course that teaches truck drivers proper livestock handling, loading, and biosecurity practices. In order to match the study conducted by González *et al.* [9] on truckloads of cattle leaving Alberta, drivers’ years of experience was split into categories according to the following criteria: <2, 2 to 5, 5 to 10 or >10 years of experience hauling cattle.

#### 2.1.2. Trailer Characteristics

Information about the trailer was gathered to assess the environment in which the cattle were transported. Drivers were asked what the make and year of their trailer was, as well as the number of trailer axles, the number of compartments being used in the trailer for the present load, and the percentage of the trailer that was covered by weather boards. The condition of the bedding at the time of unloading was scored as no bedding, dry, wet, or soupy where wet bedding was defined as the bedding being moist and clumping together, and soupy was defined as the bedding being saturated and dripping liquid. Dry bedding was defined as the bedding being loose and neither wet nor soupy. Weather boards are plastic or wood panels that can be attached to the side of the livestock trailer in order to reduce air flow into the trailer during cold weather. The use of weather boards was scored as the number of vertical strips of boarding attached to one side of the trailer.

#### 2.1.3. Trip Characteristics

Trip information gathered related to the point of origin, and the date and time of departure. The date and time of arrival at, and departure from, the rest station were also recorded. We were interested in how well trucks were meeting the requirements for maximum allowable times for animals in transit and the duration of rest outlined by Canadian federal legislation. To do this, distances travelled both to the rest station and to the final destination and time in transit were estimated and, and time spent at the rest station was calculated. Distance travelled (km), to the rest station was estimated using Google Maps [10] and the city, town or village nearest to the point of departure. The total distance to be travelled was also estimated by finding the distance between the place of origin and the city, town or village nearest to the driver’s reported final destination. The time spent in transit was estimated as the difference between the date and time of the start of the trip reported by the driver and the date and time that the truck arrived at the rest station, accounting for time zone differences. The time spent at the rest station was calculated as the difference between the dates and times of arrival at and departure from the rest station.

We recorded the type of property where the cattle were loaded as being from a ranch/farm, a feedlot or an auction market. Farms and feedlot were ultimately combined in the dataset as the truck drivers were not always able to distinguish between the two types. Lastly, weather at unloading was recorded using the temperature from a thermometer at the rest station and by scoring the weather as clear, cloudy, foggy, raining, snowing or windy. Information about the route taken was provided by the truck driver and combined with historical weather data available at Weather.org [11] to estimate the maximum and minimum temperatures during the trip. The sum of the maximum and minimum temperatures along the route was divided by two in order to obtain an estimated midpoint temperature for the trip to the rest station.

#### 2.1.4. Animal Characteristics

Information regarding the total number of animals that were on the truck as well as the number of animals in each compartment was collected. The type of animal was also recorded as beef or dairy. The estimated average weight of the animals on the truck, as reported by the driver, was recorded. As precise animal weight measures were not available for every load, approximate average animal weight was used to categorize the animals into weight classes. Weaned calves were defined as animals weighing under 300 kg, feeder calves were animals between 300 and 550 kg and market weight cattle were animals over 550 kg. Animals were also defined as cull cattle when they were over 550 kg and identified by the driver as such. The sex of the animals on a load was recorded as steers, heifers, bulls, cows, or mixed for loads containing more than one sex category.

We were interested in how well space allowances on the truck matched those outlined in the Canadian Code of Practice for the transportation of farm animals [12]. To determine this, space allowances (m^2^/animal) were calculated for each compartment of the truck by dividing the area of the compartment [13] by the number of animals in that compartment. This was then used to find the percentage deviation from the recommended stocking density for animals of that body weight [13] as defined by the Code [12]. The allometric coefficient (space allowance/[body weight]^2/3^) was also calculated for each compartment of the truck as this measure allowed for comparison between animals of different body weights [13].

The number of animals that were compromised, down or dead was recorded. Compromised is defined by the Canadian Food Inspection Agency’s (CFIA) transportation of animals program as an animal with reduced capacity to withstand the stress of transportation, due to injury, fatigue, infirmity, poor health, distress, very young or old age, impending birth or any other cause [14]. Down or non-ambulatory animals are defined in the Health of Animals Act as animals that are unable to stand without assistance or to move without being dragged or carried [2]. Truck drivers were also asked whether or not they had observed any of their animals lying down in the truck at any point in the journey.

#### 2.1.5. Animal Handling Characteristics

Information was collected on animal handling practices through video recordings and live observations during the unloading and reloading of the animals at the rest station. We recorded the time at which these processes started and ended, scored the number of slips and falls, and recorded whether the driver carried an electric cattle prod during handling. Unloading started when the first foot of the first animal stepped off the truck and ended when the last foot of the last animal stepped off the truck. Reloading started when the first foot of the first animal stepped onto the truck and ended when the last foot of the last animal stepped fully onto the truck. Time taken to unload and reload was calculated as the difference between their respective start and end times. Time taken to unload and reload was also calculated as the number of seconds per animal by dividing the time taken to unload and reload by the number of animals on the trailer.

Video recordings of both unloading and reloading were reviewed to count the number of slips and falls of animals both on the ramp to the truck and in the holding pen at the base of the ramp. Slips were counted when an animal lost its balance and had one or more of its feet slide, but still maintained an upright position and no part of the body other than the limbs came in contact with the ground. A fall was counted when an animal experienced a loss of balance such that they were no longer upright and some part of its body other than the limbs (e.g., flank) came into contact with the ground. The proportion of animals that slipped or fell was documented to compare any loads with different numbers of animals. Proportion was calculated by dividing the number of times animals slipped or fell by the number of animals in that load. This was then used to determine if the load was over or under the 1.0% level used by the American Meat Institute (AMI) transportation audit to differentiate acceptable handling [15].

Whether drivers carried an electric cattle prod was scored on a yes/no basis, with all drivers who were seen handling animals with a prod in their hand at any point during handling scoring a “yes”. It was not possible to determine whether or not the cattle prod was actually used or activated on the animals during handling. The drivers were not observed while they were on the truck and it was not clear whether the prod was on when it made contact with the animals.

### 2.2. Statistical Analysis

Descriptive statistics were run on the data using SAS 9.3 in order to find maximum, median, minimum, mean and standard deviations for all continuous variables. Frequencies and proportions were calculated for all categorical variables. Further statistics were run to provide descriptive statistics for continuous split up by weight class of animals. Variables relating to space allowance were analyzed by compartment and animal weight class.

## 3. Results and Discussion

All the results for the continuous variables that were recorded are summarized in Table 2 and results for categorical variables are summarized in Table 3. Table 4 shows results for continuous animal and animal handling characteristics that are separated by the weight class of cattle.

**Table 2:** Continuous variables collected for loads of cattle stopping for feed, water and rest near Thunder Bay, ON (TB).

Variable	Min.	Median	Max.	Mean	SD	N
Years of experience	0.00	20.00	48.00	18.68	13.97	66
Distance travelled to TB (km)	823.00	1639.00	2167.00	1566.40	306.30	129
Total distance travelled (km)	2262.00	3111.00	3833.00	3069.30	333.06	126
Time in transit to TB (h:mm:ss)	11:00:00	28:41:00	44:00:00	28:11:52	4:56:35	102
Time spent at rest station (h:mm:ss)	5:00:00	11:18:30	21:55:00	11:13:43	2:45:13	96
Temperature at unloading (°C)	−12.00	10.50	28.00	10.53	9.89	122
No. animals on truck	39.00	62.00	118.00	68.95	21.59	129
Time taken to unload (h:min:s)	0:06:12	0:14:50	0:37:41	0:16:26	0:05:48	89
Time taken to load (h:min:s)	0:07:19	0:14:45	0:37:50	0:15:42	0:05:27	99
Time to unload per animal (s)	6.31	13.61	41.50	14.29	5.54	89
Time to load per animal (s)	6.76	12.54	30.71	13.45	4.46	99
Slips unload (slips/animal/load)	0	0	0.088	0.0095	0.016	89
Slips load	0	0.016	0.22	0.023	0.034	96
Falls unload (falls/animal/load)	0	0	0.035	0.0021	0.0061	89
Falls load	0	0	0.051	0.0054	0.011	96

**Table 3:** Categorical variables collected for loads of cattle stopping for feed, water and rest near Thunder Bay, ON.

Variable	N	Category	Frequency	Proportion
Driver sex	66	Male	64	0.97
		Female	2	0.030
Driver experience	66	<2	7	0.11
		2–5	10	0.15
		6–10	7	0.11
		>10	42	0.64
CLT ^1^ training	66	Yes	16	0.24
		No	50	0.76
Trailer axles	128	Tri	122	0.95
		Tandem	6	0.047
No. compartments	128	4	25	0.20
		5	57	0.45
		6	46	0.36
Weather boards (strips)	121	0	109	0.90
		1	2	0.017
		5	7	0.058
		6	3	0.025
Bedding condition	116	Dry	56	0.48
		Wet	59	0.51
		Soupy	1	0.0086
Origin province	128	Alberta	42	0.33
		Saskatchewan	73	0.57
		Manitoba	13	0.10
Destination province	128	Ontario	113	0.88
		Quebec	15	0.12
Origin type	99	Farm	75	0.76
		Salesbarn	24	0.24
Destination type	103	Farm	75	0.73
		Slaughter	20	0.19
		Other	8	0.078
Weather at unloading	123	Clear	58	0.47
		Cloudy	49	0.40
		Foggy	1	0.0081
		Rain	13	0.11
		Windy	2	0.016
Weight class	128	Weaned	27	0.21
		Feeder	78	0.61
		Slaughter	19	0.15
		Cull	1	0.0078
		Mixed	3	0.023
Animal sex	128	Steer	104	0.81
		Heifer	17	0.13
		Cow	1	0.0078
		Bull	0	0.00
		Mixed	6	0.047
Lying down in truck	125	Yes	105	0.84
		No	20	0.16
Falls unloading	89	Acceptable (≤0.01)	79	0.89
		Unacceptable (>0.01)	10	0.11
Prod carried	102	Yes	95	0.93
		No	7	0.069

^1^ Canadian Livestock Transportation certification program.

**Table 4:** Continuous variables by weight class of loads stopping for feed, water and rest near Thunder Bay, ON.

Weight Class	Variable	Min.	Median	Max.	Mean	SD	N
Weaned	No. animals on load	79.00	98.00	118.00	100.74	10.01	27
	Time taken to unload (h:mm:ss)	0:11:23	0:18:34	0:32:47	0:19:20	0:06:18	21
	Time taken to load (h:mm:ss)	0:12:51	0:17:54	0:30:18	0:18:49	0:05:01	26
	Time to unload per animal (sec)	7.27	10.41	20.25	11.34	3.67	21
	Time to load per animal (sec)	6.76	10.75	18.74	11.25	3.05	26
	Slips unload (slips/animal/load)	0	0	0.046	0.0089	0.013	21
	Slips load	0	0.014	0.15	0.020	0.031	24
	Falls unload (falls/animal/load)	0	0	0.011	0.00094	0.0030	21
	Falls load	0	0	0.032	0.0044	0.0097	24
Feeder	No. animals on load	48.00	61.00	96.00	64.08	11.14	78
	Time taken to unload (h:mm:ss)	0:06:12	0:14:44	0:37:41	0:15:36	0:05:15	57
	Time taken to load (h:mm:ss)	0:07:19	0:13:31	0:37:50	0:14:20	0:05:11	61
	Time to unload per animal (sec)	6.31	13.89	41.50	15.10	6.08	57
	Time to load per animal (sec)	8.19	12.68	28.02	13.57	3.82	61
	Slips unload (slips/animal/load)	0	0	0.088	0.0095	0.017	57
	Slips load	0	0.017	0.22	0.026	0.036	60
	Falls unload (falls/animal/load)	0	0	0.035	0.0021	0.0063	57
	Falls load	0	0	0.051	0.0066	0.012	60
Market	No. animals on load	39.00	39.00	48.00	40.58	2.39	19
	Time taken to unload (h:mm:ss)	0:09:18	0:12:10	0:15:04	0:12:10	0:02:00	7
	Time taken to load (h:mm:ss)	0:08:22	0:14:05	0:21:30	0:14:05	0:05:34	7
	Time to unload per animal (sec)	14.31	17.38	19.93	17.02	2.05	7
	Time to load per animal (sec)	11.67	21.53	30.71	19.66	7.39	7
	Slips unload (slips/animal/load)	0	0	0.063	0.012	0.024	7
	Slips load	0	0	0.10	0.015	0.039	7
	Falls unload (falls/animal/load)	0	0	0.023	0.0063	0.011	7
	Falls load	0	0	0.021	0.0030	0.0079	7

### 3.1. Driver Characteristics

In this survey there were 66 different truck drivers who stopped at the rest station during the study period. The truck drivers had on average 18.7 ± 14.0 (mean ± SD) years of experience hauling cattle with a median of 20.0 years of experience. A majority of the drivers (63.6%) had > 10 years experience hauling cattle, which González *et al*. categorically defined as “extensive” [9]. The rest of the drivers were split fairly evenly between the experience categories: 10.2% = 5 to 10 years; 15.2% = between 2 and 5 years; 10.6% = <2 years of experience hauling cattle. There is limited research that indicates that driver experience affects the welfare of animals during transportation, however, González *et al.* [9] found that drivers with more experience had animals with less shrink at their final destination. Further, Hemsworth *et al.* [16] have documented the beneficial effects of training in improving the attitudes and behaviour of stockpersons towards dairy cattle.

Roughly one quarter (24.2% or 16) of the drivers surveyed had taken CLT training. This result is close to the 30% of drivers that Thrower [17] found had received some form of training in a study where 666 loads of feeder and yearling cattle arriving at Ontario feedlots and auction markets where surveyed. Warren *et al*. [18] surveyed 1,348 loads of market weight cattle arriving at a processing plant in Ontario in 2009 and reported that a majority of truck drivers had not received CLT training, but instead were trained on the job. Warren *et al*. [18] attributed this result to the fact that a majority of drivers surveyed were from Ontario companies, as were a majority of the drivers in this study. Although CLT training is available nationwide, it was developed and first launched by the Alberta Farm Animal Care Association in Alberta and Saskatchewan in 2007 and likely had the highest participation in those provinces, especially early after the program was launched. González *et al*. [9] found that the policy of most of the transport companies in Alberta was that all drivers must receive CLT training. According to Temple Grandin, stockperson attitude and training is the most important factor affecting animal welfare [3], however there is minimal research indicating specifically that driver experience and training affects the welfare of animals during transportation.

### 3.2. Trailer Characteristics

A majority (95.3%) of trailers in this study had three rear axles (tri-axle) with the remaining 4.7% of trailers having two rear axles (tandem). Tandem trailers have shorter back and doghouse compartments and therefore can fit fewer animals in these compartments. The number of compartments that were used ranged from four to six and varied based on the animal weight class and trailer type. All loads of weaned calves used six compartments, in both tandem and tri-axle trailers, as these animals were small enough to be able to split the nose compartment into top and bottom decks. Feeder weight animals in tri-axle trailers had the greatest variation in the number of compartments used with 12.5% of loads using four compartments, 66.7% with five compartments and 20.8% with six compartments. This difference is likely due to the variation in size and weight of these animals and whether they were too tall for the low ceilings in the nose and doghouse compartments. All loads of feeder cattle in tandem trailers had five compartments. Loads of slaughter weight animals were only observed in tri-axle trailers and 57.9% used four compartments while 42.1% used five compartments. The use of the doghouse as a fifth compartment depended on the number of animals that needed to be transported, and whether the animals were short enough to fit into the compartment. A majority of trucks surveyed used no weather boards (90.0%) while the remaining 10.0% of trailers had between 4 and 25% of the vehicle covered. As expected, all the trucks that utilized boards occurred during the months of November and December when the coldest ambient temperatures were recorded.

All of the trucks in this study were bedded with straw, which is in keeping with the Health of Animals Regulations, which state that all vehicles used to transport animals should be “littered with straw, wood shavings, or other bedding material” [2]. This result was also found in a survey of 666 loads of feeder and yearling cattle transported to auction markets and feedlots in Ontario, which found that over 99% of loads were bedded [17]. The present results differ from those found by González *et al*. [9] when they surveyed 6,152 truckloads of cattle leaving Alberta, a majority of which (89.0%) were destined for the United States. That study found only 22.7% of loads used bedding. The difference in bedding use documented by González *et al*. [9] may be due in part to the different weight classes of cattle observed. In their study, González *et al*. [9] reported that a higher proportion of loads carrying feeders and weaned calves were bedded, compared to slaughter weight cattle. They explained this difference as possibly being due to the perception of younger animals being more fragile. That study also documented a higher proportion of slaughter weight cattle than was seen in our study (82.2% *vs.* 14.8%), however all loads of slaughter weight cattle in our study were bedded. As González *et al*. [9] found that bedding was used for 97.5% of loads from companies that provided bedding for free, it is also possible that differences in bedding usage between our studies is due to different company policies on bedding fees. Bedding was reported as being wet 50.9% and dry 48.3% of the time. Only 1 load of cattle (0.86%) had soupy bedding. As expected, a higher proportion of truckloads (83.3%) with wet bedding was seen when the weather at unloading was recorded as being rainy. Weather conditions were only recorded upon arrival at the rest station and not during the whole of the trip. This means that even loads recorded as ‘clear’ may have experienced rain at some other point during the trip resulting in wet bedding from spray coming through the vents.

### 3.3. Trip Characteristics

A majority of loads (57.0%) stopping for feed, water and rest originated in Saskatchewan, followed by Alberta (32.8%) and Manitoba (10.2%). Most of the loads 88.3% were destined for Ontario, with 11.7% destined for Quebec.

We found that 75.8% of cattle came directly from a farm or feedlot, while 24.2% of cattle came from an auction market. However, this differed by season, as a higher proportion of cattle originated from auction markets in the fall months than in the spring (45.5% *vs.* 8.70%, respectively). This may be attributed to the fact that a majority (75.0%) of the weaned calves originated from auction markets and weaned calves were mainly observed being transported in the fall months. Cattle were destined for farms or feedlots for 72.8% of loads, and for slaughter in 19.4% of loads, while the remaining 7.76% were heading to other destinations, such as quarantine or auction markets.

On average, loads observed in this study stopped close to the midway point (distance) between the point of origin and their reported final destination. The average distance travelled to the point of rest was 1,566 ± 306 (mean ± SD) km with a median of 1,639 km. The average total distance trucks would ultimately travel to reach the final destination was 3,069 ± 333 km with a median of 3,833 km. Market weight cattle were transported shorter distances to the rest station (1,156 ± 330 km) compared to either feeder (1,643 ± 222 km) or weaned calves (1,625 ± 266 km). Market weight cattle were also transported shorter distances to their final destination with an average of 2,609 ± 338 km when compared to either feeder (3,166 ± 248 km) or weaned calves (3,110 ± 297 km). One possible explanation for this finding could be that producers are more concerned about the effects of long-distance transportation on shrink and meat quality when cattle are going directly to slaughter. They may believe that cattle heading to feedlots will have adequate time to recover from this stress without unmanageable economic or animal welfare costs. This may cause the producer to choose processing plants closer to the point of origin. Another possible explanation could be that there is a greater proportion of feedlots closer to Thunder Bay, than backgrounding operations or cow-calf farms. This difference could also be due to the small sample size of market weight loads, with a majority of them originating from near one of three towns in Manitoba and Saskatchewan.

The average duration of the trip to the rest station from the point of origin was 28.2 ± 5.0 hours with a maximum of 44.1 and a minimum of 11.0 hours. This indicates that all the trucks surveyed in this study were well below the maximum of 48 hours in transit when they stopped at the rest station [2]. With trucks stopping, on average, around the midway point in their journey, it is expected that none of the trucks that were surveyed would exceed 48 hours for the second half on the journey. The load that came closest to the maximum time allowed originated in Alberta and stopped to pick up cattle at four separate locations. This practice was relatively uncommon as only 7.75% (10 loads) of the observed loads picked up cattle from multiple locations, and a majority of those (8 loads) picked up cattle from only two different locations. The load with the shortest transport time originated in Manitoba and stopped at the rest station in order to make truck repairs. In support of our findings, Warren *et al*. [18] found that out of 1,348 trucks surveyed at a processing plant in Ontario, only 2 loads (0.15%) had been in transit for over 48 hours. As our study found that slaughter weight cattle were transported shorter distances, the proportion of loads that exceeded the maximum time in transit may be greater in cattle destined for feedlots. This is supported in findings by Thrower [17] who, when looking at feeder cattle arriving at auction markets and feedlots in Ontario, found that 10 of 54 trucks (18.5%) that had been in transit for over 52 hours had not stopped for feed, water and rest. This suggests that there are trucks that do not stop at the rest station, and obviously those loads were not benchmarked as part of this study population.

Every load that stopped for feed, water and rest exceeded the regulated requirement of providing a minimum of 5 hours rest. The average duration of the stay at the rest station was more than double that duration: 11.2 ± 2.8 hours with a minimum of 5.0 and a maximum of 16.8 hours. A single exception was a truck that stayed for 21.9 hours due to a mechanical issue. Previous work by where drivers self-reported the duration of their rest stop found averages of 12 hours in the study by Warren *et al*. [18] and 13 hours in the study by Thrower [17]. These long durations of rest maybe due to the regulated required rest periods for the drivers of transport vehicles, which state that drivers must rest for a minimum of ten hours per day, with at least eight hours being consecutive [19].

### 3.4. Animal Characteristics

All loads of cattle surveyed in our study were transporting beef cattle. The majority of loads were feeder cattle (60.9%) while 21.1% were weaned calves and 14.8% market weight cattle. A small number of loads 2.34% (3 loads) had both weaned and feeder weight cattle. There was only one load of cull cows surveyed (0.78%) and no loads of bulls were encountered or surveyed. The majority of loads were steers (81.3%) with 13.3% of loads heifers and 4.69% of loads with a mixture of sexes. The number of animals on the truck ranged from 79 to 118 for weaned calves, 48 to 96 for feeder weight cattle, and 39 to 48 for slaughter weight cattle. When asked whether any of their animals laid down during the trip 84.0% of drivers reported yes, that at least some animals had laid down, although this was not further quantified. Drivers were able to observe their animals lying down when they stopped during the trip and checked on their animals. 

Due to the small number of loads in tandem trailers surveyed in this study, space allowance was only calculated for tri-axle trailers. Space allowance results are summarized in Table 5 and these varied by cattle weight class and truck compartment as shown in Table 6. Cattle in the nose and doghouse compartments typically had more space than the minimum recommended with the top deck of the nose having on average 59% more space, the bottom deck of the nose 46% more space, and the doghouse 26% more space than the minimum recommended in the Canadian Code of Practice [12]. The back, belly and deck compartments were typically closer to the minimum recommended space allowance with the back compartment having on average 5.4% more space, the belly 7.2% less space and the deck 6.2% less space than the minimum. These results are consistent with those reported by González *et al*. [13]. When we discussed the extra space in the nose and doghouse compartments with truck drivers they explained that due to the smaller size and different shape of these compartments adding another animal would cause them to be too crowded, even if on paper they would be closer to the minimum stocking density. This suggests that recommendations may need to be reassessed to better reflect the usable space in these compartments. These results may be concerning as a majority (80.2%) of the cattle were transported in the back, belly and deck compartments, and were thus more likely to be exposed to overcrowded conditions.

**Table 5:** Space allowances (m^2^/head) for cattle on tri-axle trailers that were unloaded at feed, water and rest stations in Thunder Bay, ON.

Variable	Compartment	Min.	Median	Max.	Mean	SD	N
SA ^1^ (m^2^/head)	Back	0.60	1.06	1.81	1.07	0.28	116
	Belly	0.56	0.93	1.64	1.00	0.27	114
	Deck	0.59	0.94	1.64	1.01	0.26	111
	Nose bottom	0.91	1.36	2.72	1.58	0.53	112
	Nose top	0.91	1.36	1.63	1.30	0.25	40
	Doghouse	0.69	1.08	2.42	1.26	0.50	94
DRSA ^2^	Back	−0.45	0.021	0.19	0.0012	0.11	113
	Belly	−0.20	0.086	0.23	0.075	0.066	111
	Deck	−0.20	0.079	0.21	0.065	0.068	108
	Nose bottom	−1.13	−0.44	0.043	−0.47	0.26	109
	Nose top	−1.39	−0.611	−0.14	−0.60	0.23	38
	Doghouse	−1.16	−0.18	0.16	−0.23	0.26	90
AC ^3^	Back	0.015	0.019	0.027	0.019	0.0021	113
	Belly	0.014	0.018	0.022	0.018	0.0014	111
	Deck	0.015	0.018	0.022	0.018	0.0014	108
	Nose bottom	0.018	0.027	0.040	0.028	0.0050	109
	Nose top	0.021	0.030	0.044	0.030	0.0044	38
	Doghouse	0.016	0.022	0.042	0.023	0.0053	90

^1^ Space allowance (m^2^/head); ^2^ Deviation from recommended space allowance ((recommended-observed)/recommended); ^3^ Allometric coefficient (space allowance/(body weight)^2/3^).

**Table 6:** Space allowances (m^2^/head) for cattle on tri-axle trailers that were unloaded at feed, water and rest stations near Thunder Bay, ON by weight class.

Variable	Weight Class	Compartment	Min.	Median	Max.	Mean	SD	N
SA ^1^	Weaned	Back	0.60	0.74	0.97	0.75	0.090	29
		Belly	0.56	0.70	0.82	0.69	0.069	27
		Deck	0.59	0.70	0.83	0.70	0.066	26
		Nose bottom	0.91	1.17	1.63	1.15	0.19	27
		Nose top	0.91	1.17	1.63	1.20	0.22	27
		Doghouse	0.69	0.81	1.08	0.80	0.10	27
	Feeder	Back	0.79	1.06	1.41	1.08	0.15	67
		Belly	0.73	1.04	1.23	0.99	0.13	67
		Deck	0.76	1.04	1.23	1.00	0.12	65
		Nose bottom	0.91	1.63	2.04	1.50	0.29	64
		Nose top	1.02	1.63	1.63	1.51	0.20	12
		Doghouse	0.81	1.38	2.42	1.35	0.38	58
	Market	Back	1.27	1.58	1.81	1.53	0.15	19
		Belly	1.31	1.41	1.64	1.46	0.11	19
		Deck	1.31	1.41	1.64	1.46	0.96	19
		Nose bottom	1.63	2.72	2.72	2.52	0.36	19
		Nose top	-	-	-	-	-	-
		Doghouse	1.94	2.43	2.43	2.24	0.25	8
DRSA ^2^	Weaned	Back	−0.33	0.057	0.19	0.019	0.12	29
		Belly	−0.020	0.099	0.23	0.085	0.081	27
		Deck	−0.20	0.089	0.16	0.067	0.090	26
		Nose bottom	−1.00	−0.45	−0.14	−0.52	0.20	28
		Nose top	−1.39	−0.53	−0.14	−0.58	0.26	26
		Doghouse	−0.58	0.0014	0.10	−0.049	0.15	27
	Feeder	Back	−0.45	0.014	0.15	−0.0068	0.10	65
		Belly	−0.051	0.084	0.21	0.080	0.057	65
		Deck	−0.072	0.073	0.21	0.070	0.057	63
		Nose bottom	−1.13	−0.36	0.043	−0.40	0.27	62
		Nose top	−0.79	−0.75	−0.20	−0.66	0.18	12
		Doghouse	−1.16	−0.22	0.16	−0.28	0.26	55
	Market	Back	−0.24	0.0045	0.11	0.0016	0.097	19
		Belly	−0.037	0.039	0.17	0.044	0.066	19
		Deck	−0.079	0.080	0.12	0.044	0.66	19
		Nose bottom	−0.91	−0.73	−0.25	−0.64	0.18	19
		Nose top	-	-	-	-	-	-
		Doghouse	−0.86	−0.52	−0.29	−0.50	0.19	8
AC ^3^	Weaned	Back	0.015	0.018	0.024	0.018	0.0022	29
		Belly	0.014	0.017	0.022	0.017	0.0015	27
		Deck	0.016	0.017	0.022	0.017	0.0016	26
		Nose bottom	0.021	0.027	0.037	0.028	0.0037	28
		Nose top	0.021	0.028	0.044	0.029	0.0047	26
		Doghouse	0.017	0.019	0.029	0.019	0.0027	27
	Feeder	Back	0.016	0.019	0.027	0.019	0.0019	65
		Belly	0.015	0.018	0.020	0.018	0.0011	65
		Deck	0.015	0.018	0.020	0.018	0.0011	63
		Nose bottom	0.018	0.026	0.040	0.027	0.0051	62
		Nose top	0.022	0.033	0.034	0.031	0.0034	12
		Doghouse	0.016	0.023	0.042	0.025	0.0050	55
	Market	Back	0.017	0.020	0.025	0.020	0.0019	19
		Belly	0.017	0.019	0.021	0.019	0.0013	19
		Deck	0.018	0.018	0.021	0.019	0.0013	19
		Nose bottom	0.024	0.034	0.038	0.033	0.0037	19
		Nose top	-	-	-	-	-	-
		Doghouse	0.026	0.030	0.036	0.030	0.0036	8

^1^ Space allowance (m^2^/head); ^2^ Deviation from recommended space allowance; ^3^ Allometric coefficient (space allowance/(body weight)^2/3^).

Market weight cattle had the highest values for space allowance compared to weaned and feeder cattle, as shown by the larger allometric coefficient for these animals across compartments. These results are consistent with those reported by González *et al*. [13]. This was explained by González *et al*. [13] as being the result of limitations on the amount of weight allowable per axle by Ministry of Transportation regulations. With larger animals there is more weight per square metre and with slaughter weight cattle, the number of animals that can be loaded is more often limited by weight regulations than by stocking density. Therefore, market weight cattle are often loaded with a higher space allowance (less crowded) than smaller cattle.

Transporting cattle at stocking densities exceeding the values recommended in the Canadian Code of Practice [12] has been shown to result in significantly increased levels of bruising and muscle damage [8], which is both an issue for the well-being of cattle and the person marketing them. It was suggested by Tarrant *et al*. [8] that more animals fall down at higher stocking densities and also that animals that fall or lie down during the journey at high stocking densities may not be able to stand-up easily and may be trampled, resulting in increased bruising.

Results for the number of compromised, down or dead cattle on each load are summarized in Table 7. No cattle were dead on arrival at the rest station for any loads surveyed in this study. Just one load (0.96% of all loads) contained an animal recorded as down, and 2.88% of loads had an animal that was deemed to be compromised (3 loads). Overall, there were three animals (0.040%) that were lame at unloading and one animal (0.013%) that was down on the trailer, for a total of four (0.053%) compromised animals. Compromised animals observed in this study were unloaded and kept in a separate pen for the duration of their stay at the rest station. When it was time to reload they were assessed for fitness for transport and in all cases those animals were left at the rest station and later euthanized. Having animals on the truck that are compromised, down or dead is an obvious welfare issue and perhaps a likely an indicator of poor transportation practices. The AMI transportation audit program developed by Temple Grandin assesses the welfare of animals, including cattle, arriving at processing plants in the United States. This audit uses a level of 1.9% of cattle on the truck being compromised or down as acceptable [15]. Every load in our study was within the acceptable limits outlined by the AMI transportation audit [15]. All of the cases of compromised animals in this survey were due to lameness. The proportion of compromised animals seen in this study were either similar to, or lower than, those seen in other studies. The percentage of lame animals was similar to the 0.022% reported by González *et al*. [9] and the 0.065% reported by Thrower [17]. Warren *et al*. [18] reported higher percent of lame animals at 0.16%. One possible reason for this higher proportion of lameness could be that the researchers used a more sensitive definition for lameness. However, lameness was not operationally defined in their paper and so this could not be verified as the cause. Another explanation could be that lameness is more prevalent among market weight cattle arriving at processing plants than in the feeder and weaned calves that were predominant in our study. The percentage of animals that were down, or needed assistance was similar in our study (0.013%) to the 0.002%–0.025% reported by the others [9,17,18]. No dead animals were seen in this study, but this may be due to the present sample size of 8,894 animals when compared to the other studies that have looked at this variable, which have found 0.008%–0.015% of animals dead had sample sizes of 19,977–290,362 animals [9,17,18].

**Table 7:** Number of compromised, down, and dead cattle observed for loads stopping for feed, water and rest in Thunder Bay, ON, based on their production classes. No load contained more than one compromised, down, or dead animal.

Weight Class	Compromised ^1^	Down ^2^	Dead	Loads ^3^	Animals ^4^
Weaned	1	1	0	27	4,154
Feeder	2	0	0	65	2,720
Market	0	0	0	7	300
Other	0	0	0	5	405
All	3	1	0	104	7,579

^1^ Defined as an animal with reduced capacity to withstand the stress of transportation, due to injury, fatigue, infirmity, poor health, distress, very young or old age, impending birth or any other cause; ^2^ Defined as an animal that is unable to stand without assistance or to move without being dragged or carried; ^3^ Number of truckloads of animals observed; ^4^ Number of individual animals observed.

When the characteristics of loads with compromised or down animals were examined we found that all of the affected loads arrived at the rest station during the month of October. This might indicate that season plays a role in the probability of an animal becoming compromised or down during transport and could be an area for future research. The single load with an animal down in the trailer had mixed steers and heifers within the belly compartment, where the animal was down. This may have caused problems due to differences in size between steers and heifers, although we did not quantify this directly. The mixing of sexes may have caused increased activity within the compartment leading to one of the animals getting injured. However, Yeh *et al*. [20] did not find any increase in bruising when mixing sexes of cattle on a transport truck. It was noted by both the researcher (Hannah Flint) and the rest station manager that the compartment had a couple of animals, including the one that was down, that were noticeably smaller than the rest. Variability in the size of animals within compartments was not measured in this study, but could be a contributing factor in incidence of compromised or down animals. One of the loads with a compromised animal due to lameness had the longest time in transit (44 hours). As studies have shown that injuries increase with journeys of longer duration [6,21] it is possible that the long time in transit was a contributing factor to the lameness seen in that particular load. Other loads with compromised animals had no measured unique characteristics that in any way seemed to set them apart from other loads.

### 3.5. Animal Handling Characteristics

In this study, it took on average 16.4 ± 5.81 (mean ± SD) minutes to unload and 15.7 ± 5.45 minutes to load cattle. The time taken to unload in our study was less than the 30 minutes reported by González *et al*. [9]. One reason for this difference could be due to differences in facility design. González *et al*. had unloading times from various facilities. This difference in time could also be due to variation in handling techniques, or differences in speed in market weight animals observed in their study, compared to predominantly feeder weight animals observed in our study. This last possibility is supported by the fact that we saw longer unloading times in market weight animals when the number of animals on the truck was accounted for. The time taken to unload was greater than the 8.4 minutes taken to unload cattle in Europe reported by Villarroel *et al*. [22]. The reason for this difference could be due to the fact that the average number of animals on the trucks in Europe was much lower (16 *vs.* 69) than the present study, and the majority of the trucks in the study from Europe were small, two-axle, vehicles designed to carry eight animals and had fewer compartments than the trailers we observed [22]. When time taken to unload per animal was calculated the time of 14.29 seconds per animal seen in our study was roughly half of the 31.50 seconds calculated from the study conducted by Villarroel *et al*. [22]. The unloading time per animal seen in our study was also much less than the 72.0 seconds reported by Maria *et al*. [23], which like the study conducted by Villarroel *et al*. [22] was conducted in Europe. Differences in trucks and handling techniques in Europe may result in longer unloading times. The time to load in our study was similar to the 20.0 minutes reported by González *et al*. [9] and 16.8 minutes reported by Villarroel *et al*. [22]. However, when the time to load per animal was calculated the 13.45 seconds per animal seen in our study was considerably less than the 63.00 seconds found by Villarroel *et al*. [22] and 86.0 seconds found by Maria *et al*. [23]. This could be due to differences in the amount time needed to open and close dividers in European trucks compared to those used in North America. Loading and unloading can be the most stressful period during transportation [4]. Thus, it could be argued that a faster loading and unloading time is beneficial to the animals as it reduces the time spent being handled. This is supported by the fact that Maria *et al*. [23] found trucks that experienced shorter unloading times (under 1 minute) had animals with lower levels of cortisol, creatine kinase and lactate at slaughter when they also had fewer slips, falls and balks. Conversely, it could indicate that the cattle are rushing off of, or onto, the truck in order to escape a negative environment or handling stimuli, which we attempted to control for/quantify by counting the number of slips and falls. The data collected here does not suggest the truck is a negative environment for the cattle as the time to load was less than the time to unload, indicating that the cattle are returning to the trailer easily. However there is the possibility that cattle may be moving quickly to escape negative handling stimuli when being loaded, despite potentially finding the truck environment aversive. One limitation to using the gross duration of unloading and reloading as a measure of handing is that it included time the driver spent opening and closing compartments, moving bedding, counting animals (in some cases recounting animals), *etc*. This means the actual ‘hands-on’ animal handling time is likely less than what we recorded.

In this study, an average of 0.74% of cattle slipped in each load during unloading and 0.16% fell with a maximum of 8.77% and 3.51% respectively. More cattle slipped and fell during reloading with an average of 2.26% of cattle in each load slipping during reloading and 0.44% falling, with maximums of 22.0% and 5.08% respectively. Under the AMI transportation audit program the number of cattle that fall during unloading must be less than 1% in order to be deemed acceptable [15]. When the AMI cut off of 1% is applied to the loads surveyed here, 89.0% of all loads would be deemed acceptable. Slips and falls during unloading and reloading can be an indicator of improper facilities or handling techniques as cattle may be rushing to avoid a negative stimulus or may not have proper footing [3]. In order to further assess the behaviour and welfare of cattle during unloading and reloading the speed of cattle movement, or number of balks could additionally be recorded.

Truck drivers were seen holding a cattle prod during either unloading or reloading in 93.1% of loads, but it was not possible to record whether the prod was being activated by the handlers as it some do touch the cattle with the prod as they count them, without applying any shock. Huertas *et al*. [6] found that the use of devices that force cattle to move, such as a cattle prod, during loading or unloading en route to processing plants increased the level of bruising seen in the carcasses. Even if the prod was not used here, it has been suggested when an electric prod is not the primary driving tool worker attitude improves and handlers are less likely to yell at or hit the animals [3]. Some drivers stated they felt the need to carry a cattle prod during loading to ensure their personal safety, which may suggest that there is a need for more training on other, more welfare-friendly, driving tools.

A major limitation of the present sample is that only trucks that stopped for feed, water and rest at the studied rest station were surveyed and thus, the results are limited to drivers and companies that are actually operating under compliance with the regulations. This study did not include loads that chose to stop at the other commercial rest station near Thunder Bay, which may have a different demographic of clientele. There may be loads that legally should be stopping for rest, but that disobey the laws and regulation. To further assess how well all cattle being transported long-distances in Canada are coping, further studies that include loads from the other rest station near Thunder Bay, as well as loads that do not stop for feed, water and rest should be examined.

## 4. Conclusions

This study is the first comprehensive examination of loads of commercial cattle being transported long distances within Canada. Results confirmed that a majority of the loads stopping for feed, water and rest were calves destined for further feeding (82.0%). Loads were being rested after 28.2 ± 5.0 hours on the road, well under 48 hours, which is the maximum allowed by law. Additionally the loads observed offered, on average, 11.2 ± 2.8 hours of rest, which is more than double the minimum of 5 hours legislated. These results indicate the loads observed were following the regulations stated by Canada’s Health of Animals Act. However, there is variability around how closely recommendations, such as stocking density, were being followed. The results relating to stocking density indicate that it may be beneficial to update the recommendations to provide stocking densities for different compartments to reflect differences in usable space. In order to assess how well cattle themselves are coping under the current transportation regulations and practices, further research into how these factors affect the cattle needs to be undertaken. Further research should also be conducted to determine how many loads fail to stop for feed, water and rest and how this transgression of the regulations might be affecting the health and welfare of cattle transported under those conditions.
